# Altered Expressions of *IL-15*, *IFNG*, and *HPRT1* Genes in the Thin Endometria of Patients with Reproductive Disorders: A Prospective Comparative Study

**DOI:** 10.3390/jcm13206184

**Published:** 2024-10-17

**Authors:** Almagul Kurmanova, Yeldar Ashirbekov, Gaukhar Kurmanova, Nagima Mamedaliyeva, Gaini Anartayeva, Gaukhar Moshkalova, Damilya Salimbayeva, Aidana Tulesheva, Zhamilya Zhankina

**Affiliations:** 1 Faculty of Medicine and Healthcare, Al Farabi Kazakh National University, 71, Al-Farabi Avenue, 050040 Almaty, Kazakhstan; 2 Laboratory of Structural and Functional Genomics, M. Aitkhozhin Institute of Molecular Biology and Biochemistry, 86, Dosmukhamedov Street, 050012 Almaty, Kazakhstan; 3 Department of Science and Strategy, Scientific Center of Obstetrics, Gynecology and Perinatology, 125, Dostyk Ave., 050010 Almaty, Kazakhstan; 4 Faculty of Natural Sciences, Friedrich Alexander University Erlangen Nürnberg, Schlossplatz 4, 91054 Erlangen, Germany

**Keywords:** endometrial receptivity, recurrent implantation failure, recurrent pregnancy loss, thin endometrium, transcriptomics

## Abstract

Reproductive disorders are common events in modern reproductive medicine, occurring both in spontaneous and assisted pregnancies. Studies on the molecular mechanisms of implantation disorders in thin endometria, including the study of gene transcriptional activities, have shed light on the identification of the potential biological markers of endometrial receptivity. **Background/Objectives**: The goal of this study was to reveal the significantly dysregulated selected gene expressions between RIF and RPL patients with thin endometria. **Methods**: Endometrial samples were collected from RIF patients (*n* = 20) and RPL patients (*n* = 19) during the implantation window days (LH + 7—LH + 10) of their natural menstrual cycles. Ten genes were chosen as the target genes regarding their possible relations with the implantation process. The total RNA was purified and reverse-transcribed, and gene expressions were quantified by RT-PCR. **Results**: The expressions of the *IL-15*, *INFG*, and *HPRT1* genes were significantly decreased in the RIF patients with thin endometria compared to the RPL patients (log_2_ fold change = 0.92, *p* = 0.023 for *IL-15*; log_2_ fold change = 1.24, *p* = 0.046 for *INFG*; and log_2_ fold change = 0.579, *p* = 0.046 for *HPRT1*). There were no significant differences in the expressions of the *CXCL8*, *CXCL1*, *MMP10*, *C4BPA*, *TNC*, *VEGFB*, and *HAND2* genes between the groups. **Conclusions**: Decreased expressions of the *IL-15, INFG*, and *HPRT1* genes were found in patients with RIF with thin endometria compared to the endometria of women with RPL. This has practical significance for clinicians for the differentiated prescription of immunomodulatory therapy in patients undergoing ART programs.

## 1. Introduction

Reproductive disorders are commonly seen in modern reproductive medicine, occurring both in spontaneous and assisted pregnancies [1]. Successful implantation requires a good-quality embryo, a receptive endometrium, and synchrony between the two. Despite advances in assisted reproductive technology (ART) programs in terms of improving the embryo quality, including preimplantation genetic screening, the causes of adverse reproductive outcomes are multifactorial, and the endometrial factor remains the main factor in low IVF success rates, as abnormal endometrial receptivity accounts for two-thirds of implantation failures [2].

A receptive endometrium is not in a binary state (all or nothing); rather, the degree and type of abnormal receptivity lead to a range of reproductive problems, spanning from complete recurrent implantation failure (RIF) or infertility to severe pregnancy loss (RPL) or miscarriage and mild implantation failure and invasion (preeclampsia) [3]. Despite the importance of endometrial receptivity for implantation, the precise mechanisms involved in the regulation of endometrial receptivity remain poorly understood [4].

Abnormal receptivity is also associated with a thin endometrium (an endometrial thickness of less than 7 mm during the “implantation window”), and the exact etiology and physiopathology of this condition remain largely unclear [5,6]. Existing treatments are unable to achieve satisfactory clinical effects in many patients with thin endometria [7]. Studies of the molecular mechanisms of implantation disorders in thin endometria, including the study of gene transcriptional activity, have shed light on the identification of the potential biological markers of endometrial receptivity [8,9,10].

In the receptive phase of the endometrium in a healthy woman, a large number of genes, representing various signaling pathways and meta-signatures, are differentially expressed [11,12]. However, many researchers have proposed the use of the most frequently occurring genes for further analysis [13,14]. Gene ontology analyses have revealed that widespread meta-signatures are represented by immune response genes, including the pro-inflammatory signaling cascade (*C-X-C motif chemokine ligand 8 (CXCL8)* and *C-X-C motif chemokine ligand 1 (CXCL1*)), the complement cascade (*complement component 4 binding protein alpha (C4BPA))*, the abnormal activation of the innate and adaptive immune systems (*interleukin 15 (IL-15)*, *heart- and neural-crest-derivative-expressed transcript 2 (HAND2),* and *interferon gamma (INFG))*, vascular proliferation (*vascular endothelial growth factor B (VEGF-B))*, the breakdown of the extracellular matrix and tissue remodeling (*matrix metallopeptidase 10 (MMP10*)), the generation of purine nucleotides (*hypoxanthine phosphoribosyltransferase 1 (HPRT1*)), and cell adhesion (*tenascin C (TNC))* [15,16,17,18,19].

In this study, we took 10 of the above-mentioned genes (*CXCL8*, *CXCL1*, *HPRT1*, *MMP10*, *INFG*, *C4BPA*, *TNC*, *VEGFB*, *HAND2*, and *IL15*) that showed altered activities in the receptive phases in healthy women. Bioinformatics allows for selecting genes with up- and downregulated expressions in the receptive endometrium phase [11,12]. In studies devoted to the study of gene meta-signatures in the implantation windows in healthy women, the groups of genes responsible for endometrial proliferation [15] and immune response [18,19] are widely represented. Therefore, the selection of genes involved in these processes can provide information on disturbances in endometrial receptivity processes.

The research questions were whether the expressions of the selected genes would differ in thin endometria with clinical forms of implantation disorders (RIF and RPL), and if there were differences, then, in which direction should therapeutic approaches be developed?

## 2. Materials and Methods

### 2.1. Subjects

This study was conducted among the women who applied to the Scientific Center of Obstetrics, Gynecology, and Perinatology (Almaty, Kazakhstan) for reproductive disorders from September to December 2023. The inclusion criteria for the group were the presence of a thin endometrium (less than 7 mm during the implantation window, as measured during an ultrasound examination) and a history of reproductive losses in anamnesis (RPL or RIF).

RPL was defined based on the ESHRE guidelines of having had 2 or more miscarriages confirmed by a positive human chorionic gonadotropin test [20]. RIF was defined as having had at least 3 unsuccessful transfers of good-quality embryos after in vitro fertilization–embryo transfer [21]. In addition, the women’s age was <40 years, and they did not have any karyotype anomalies.

A total of 39 patients were recruited for transcriptional analysis (20 RIF and 19 RPL patients).

### 2.2. Ethics Approval

This study was approved by the Ethical Committee of Al Farabi Kazakh National University, Kazakhstan (Code: IRBA400/IRB 00010790). All the patients have been asked to sign a written informed consent.

### 2.3. Sample Processing

Endometrial samples were obtained by pipelle biopsies using a Goldstein catheter (SonoBiopsy^TM^, J-GSBX-072026, size (Fr) 7.2, Cook Incorporated, Bloomington, IN, USA) in LH + 7 − LH + 10 days (the implantation window) of the natural menstrual cycle. Samples from RPL (*n* = 19) and RIF patients (*n* = 20) with thin endometria were transferred to cryotubes with 1 mL of RNA-later stabilization solution (Thermo Fisher Scientific, Vilnius, Lithuania) and stored in a refrigerator at 4 °C for 12 h and the next day, stored in a freezer at −20 °C. After the sample collection was completed, the samples were transferred to the M. Aitkhozhin Institute of Molecular Biology and Biochemistry (Almaty, Kazakhstan).

### 2.4. RNA Isolation from Endometrial Samples

The isolation of the total RNA from the endometrial samples was performed using a Dynabeads™ mRNA DIRECT™ purification kit (Thermo Fisher Scientific) according to the manufacturer’s protocol. The isolated RNA was immediately subjected to further analysis.

### 2.5. cDNA Synthesis and Quantitative Polymerase Chain Reaction (PCR)

Reverse-transcription and quantitative PCR were performed using primers and probes from a TaqMan™ gene expression assay (Applied Biosystems, Vilnius, Lithuania). cDNA was obtained using a high-capacity cDNA reverse-transcription kit (Applied Biosystems) according to the manufacturer’s protocol. Quantitative PCR was performed in duplicate using TaqMan’s fast advanced master mix (Applied Biosystems), under the conditions recommended by the manufacturer, and a StepOnePlus real-time PCR system (Applied Biosystems). Primary processing was performed using StepOnePlus 2.2.2 and ExpressionSuite v.1.3 programs. The relative quantification of the gene expressions was carried out using the comparative Ct (ΔΔCt) method with modifications, as described by Königshoff M. et al. (2009) [22]. The relative transcript abundances are expressed in ΔCt values (ΔCt = Ctreference − Cttarget). *Glyceraldehyde-3-phosphate dehydrogenase (GAPDH)* and *tyrosine-3-monooxygenase/tryptophan-5-monooxygenase activation protein zeta (YWHAZ)* housekeeping genes were used as references, in accordance with a previous study [11]. The ΔΔCt value (ΔΔCt = ΔCtcase − ΔCtcontrol) was considered as a log2 fold change.

### 2.6. Statistical Analysis

Most of the statistical analyses were performed in the Jamovi program (https://www.jamovi.org, accessed on 5 September 2024). The statistical significances of the differences in the ΔCt values between the groups were calculated using the two-tailed Mann–Whitney U test. Spearman’s rank correlation method was used to examine the relationships between the quantitative variables, and *p* < 0.05 was considered as statistically significant. Because of the explorative nature of this study, no adjustments for multiple testing were performed. For a comparative visualization of the gene expression levels, boxplots were constructed using a web tool, BoxPlotR (http://shiny.chemgrid.org/boxplotr, accessed on 5 September 2024). The characteristics of the markers were evaluated by ROC analysis using the web tool easy ROC v.1.3.1 [23] and Jamovi. Youden’s index method was used to calculate the optimal cut-off points. The evaluation of classifiers by the interpretation of the area under the ROC curve (AUC) was performed as described by [24].

## 3. Results

### 3.1. Demographic and Clinical Characteristics of the Study Groups

The demographic and clinical characteristics of the study groups are presented in Table 1.

After the clinical characteristics were compared between the two groups, no statistical differences were found by age and BMI. All the participants were under 40 years of age. The prothrombin time was significantly reduced in the RIF group (*p* < 0.05), which indicates a patient’s tendency to have thrombotic vascular lesions. There were no significant differences between the groups in blood hormone levels.

### 3.2. The Levels of mRNA in Endometrial Samples of RIF Patients in Comparison with RPL Patients

Comparative gene expression statistics between RPL and RIF patients are presented in Table 2.

**Figure 1:**
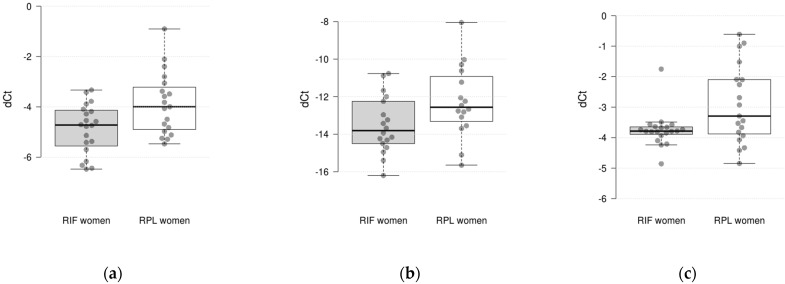
Differences in gene expression levels (ΔCt values) in endometrial tissues between RIF and RPL patients: (**a**) *IL15* gene; (**b**) *IFNG* gene; (**c**) *HPRT1* gene.

This study showed that the expressions of 3 out of 10 genes differed significantly between the studied groups. It was found that the expressions of the *IL-15, INFG*, and *HPRT1* genes were significantly decreased in RIF with thin-endometrium patients compared to RPL patients (log_2_ fold change = 0.92, *p* = 0.023 for *IL-15*; log_2_ fold change = 1.24, *p* = 0.046 for *INFG*; and log_2_ fold change = 0.579, *p* = 0.046 for *HPRT1*) (Figure 1). There were no significant differences in the expressions of the *CXCL8, CXCL1, MMP10, C4BPA, TNC, VEGFB*, and *HAND2* genes between the groups.

### 3.3. Associations with Clinical and Laboratory Characteristics

Spearman’s rank correlation method was used to examine the relationships between the expressions of the studied genes and quantitative laboratory characteristics in the two groups. The correlations are presented in Table 3. In patients with RIF, significant inverse moderate correlations were observed between *HAND2* and *TSH* (rho = −0.671, *p* = 0.001), *HAND2* and fibrinogen (rho = −0.465, *p* = 0.038), *VEGFB* and prolactin (rho = −0.446, *p* = 0.049), and *VEGFB* and *TSH* (rho = −0.526, *p* = 0.017). In patients with RPL, significant inverse moderate correlations have been established between *CXCL1* and the prothrombin time (rho = −0.456, *p* = 0.04959) and between *MMP10* and the activated partial thromboplastin time (rho = −0.494, *p* = 0.0317) and a direct moderate correlation between *CXCL1* and the activated partial thromboplastin time (rho = 0.587, *p* = 0.0083). It should be noted that after adjustment for multiple comparisons, none of the correlations remained significant.

### 3.4. ROC Analysis

**Figure 2:**
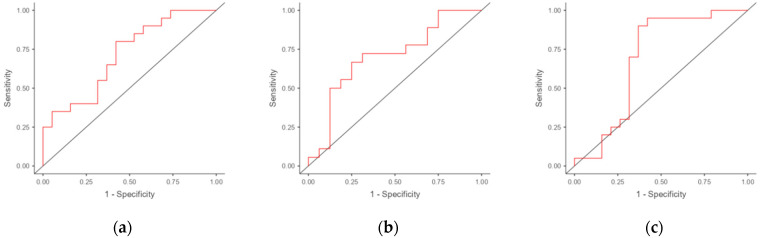

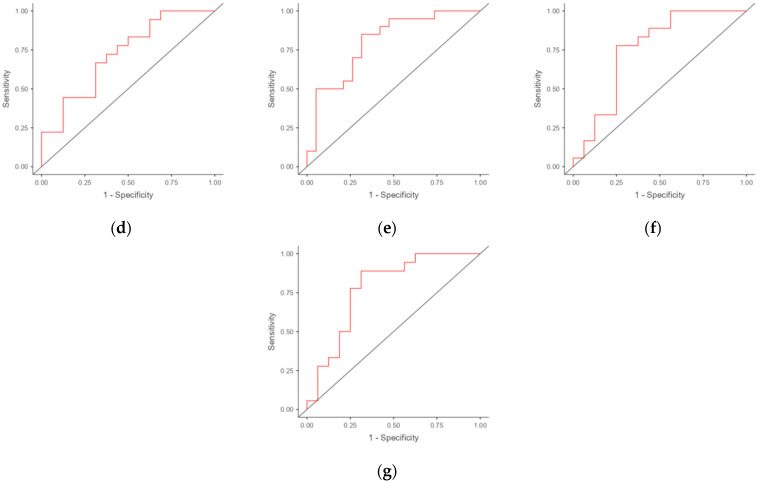
ROC curves for potential markers and combinations of markers in the discrimination of RIF and RPL in women. The ROC curves (red lines) allow for the evaluation of the quality of the binary classifications by displaying the relationship between sensitivity and specificity as the decision threshold is varied: (**a**) *IL15*; (**b**) *IFNG*; (**c**) *HPRT1*; (**d**) combination of *IL15* and *IFNG*; (**e**) combination of *IL15* and *HPRT1*; (**f**) combination of *IFNG* and *HPRT1*; (**g**) combination of *IL15*, *IFNG*, and *HPRT1*.

We performed ROC analysis to evaluate the potential for using the mRNA of genes differentially expressed in the endometria of RIF patients as markers for predicting RIF. The results are presented in Table 4. The areas under the ROC curves (AUCs) were obtained for *IL-15* (0.713), *INFG* (0.701), and *HPRT1* (0.692) (Figure 2). The best combination was the combination of *IL-15* and *HPRT1*, which resulted in an increase in the AUC to 0.800 (with a specificity of 68.4% and a sensitivity of 85.0%).

## 4. Discussion

Using the Kyoto Encyclopedia of Genes and Genomes and Gene Ontology databases for the functional enrichment of genes has yielded significant results in reproductive medicine [17,25]. Endometrial receptivity in healthy women has been associated with the upregulation of several genes in the receptive phase of the endometrium [26,27,28,29,30], including angiogenesis [15], metabolism, the extracellular matrix, tissue remodeling [17], the immune response [18,19], and cell adhesion [31].

Endometrium-level dysregulation in patients suffering from implantation disorders (i.e., RIF or RPL) has functional similarities [25]. Simultaneously, patients with RIF have a defect in endometrial receptivity that alters the embryo–endometrium dialog and prevents implantation; meanwhile, RPL is characterized by optimal receptivity with a defect in embryo selectivity, allowing for abnormal embryos to implant and increasing the risk of miscarriage [32]. Women with RIF often have imbalances in key cytokines, such as interleukin-1 (IL-1), interleukin-6 (IL-6), and tumor necrosis factor-alpha (TNF-α). The mechanisms leading to RIF may be either the depletion of cytokines required for apposition–adhesion or excess cytokines, leading to local cytotoxicity that may disrupt embryonic implantation [18]. In contrast, RPL is associated with excess amounts of inflammatory cytokines. This hyperinflammatory environment may promote the implantation of abnormal embryos because of the inability to selectively recognize embryo viability. Elevated IL-6 and TNF-α levels are associated with adverse pregnancy outcomes, supporting the notion that an inflammatory environment may adversely affect pregnancy [32].

Proper decidualization is essential for the successful implantation and maintenance of pregnancy [33]. In women with RIF, inadequate decidualization may result from a hormonal imbalance (progesterone resistance), resulting in an underprepared endometrium that cannot support embryo implantation. In RPL, although the endometrial lining appears to be receptive, impaired decidualization may be due to underlying genetic or epigenetic factors that influence trophoblast invasion and maternal–fetal interactions [34].

Successful implantation also depends on adequate angiogenesis [35]. In RIF, there may be inadequate vascular remodeling because of the altered expressions of angiogenic factors. This may result in decreased blood flow and nutrient supply to the developing embryo. Conversely, although angiogenesis may be adequate in RPL, it may be impaired in RIF. Excessive vascular permeability and inflammation can create a hostile environment for the embryo, contributing to pregnancy loss.

Our study is devoted to how significant the differences are in gene expression in the thin endometrium between RIF and RPL patients. In this study, gene expressions in RIF patients with thin endometria, along with matched endometrial tissues from RPL patients, were explored. We revealed the abnormal activation of the inflammatory environment in the thin endometrium. Immune response changes at the endometrial level were statistically significantly impaired in the RIF group. The expressions of the *IL-15*, *INFG*, and *HPRT1* genes were significantly decreased in RIF patients with thin endometria compared with RPL patients (*p* = 0.023, *p* = 0.046, and *p* = 0.046, respectively).

IL-15 is a key immune factor required for the activation and survival of uterine natural killer (uNK) cells. Activated uNK cells can promote spiral artery remodeling and secrete cytokines to induce immunotolerance [19]. *IL-15* expression is different in patients with RIF compared with fertile controls and correlates with local uNK (CD56+) recruitment and angiogenesis. Moreover, *IL-15* mRNA levels in the mid-luteal endometrium were positively correlated with CD56+ uNK cells (r = 0.392; *p* = 0.008) [36].

In our study, the downregulation of the *interferon gamma (INFG)* gene in RIF patients with thin endometria indicated NK cell deficiency. NK cells are the main source of *IFNG* production [37]. The dysregulated expressions of the IFNG gene and protein can provide critical insights into the implantation failures observed in women with RIF and RPL. Endometrial receptivity is tightly regulated during the implantation window, with a specific balance of cytokines, including IFNG. Studies have shown that IFNG plays critical roles in modulating the immune response and promoting the decidualization process. In women with RIF, particularly those with thin endometria, the downregulation of the IFNG gene expression can indicate a deficiency in NK cells, which are the primary producers of IFNG. A recent study [38] has shown that the peripheral blood level of IFNG in the RPL group was significantly higher than that in the RIF group (*p* < 0.05).

The timing of the sample collection is essential. If samples are taken during the secretory phase, when implantation typically occurs, the expression of the IFNG should peak to facilitate an appropriate immune environment. Studies have reported decreased IFNG levels during this critical period in women with RIF, which align with findings of thin endometria and NK cell dysfunction. Conversely, studies focusing on RPL have often reported higher peripheral blood levels of IFNG, especially in early gestation (up to 6–8 weeks). This discrepancy highlights that RIF may involve insufficient IFNG production, leading to impaired implantation, while RPL might involve an initial immune response that is overly aggressive or misdirected, potentially contributing to the loss of viable pregnancies.

A decrease in INFG production indicates the presence of acute and chronic viral diseases [39]. Chronic endometrial infections may cause a persistent inflammatory state, altering cytokine profiles, including IFNG downregulation. This state results in a breakdown of the immune tolerance required for implantation. A history of recurrent infections in women with RIF/RPL may result in cumulative immune changes. Furthermore, repeated infections may promote autoimmunity development, leading to inappropriate responses to both embryonic and maternal tissues. In summary, aberrant IFNG gene and protein expressions in women with RIF and RPL correlate with specific gestational ages, highlighting differences in endometrial immune responses and susceptibility. IFNG downregulation in patients with RIF and thin endometria indicates important roles for NK cells and their dysfunction. In contrast, the higher IFNG levels observed in RPL may indicate an overly aggressive immune response. Clearing infections and restoring a balanced immune response may be critical for improving outcomes in women experiencing RIF and RPL. This indicates a significant pathogenetic mechanism of implantation failure associated with infection, which must be considered in the rehabilitation of patients with RIF.

This study demonstrated the downregulation of the *hypoxanthine phosphoribosyltransferase 1 (HPRT1)* gene in RIF patients. The protein encoded by this gene is a transferase that plays a central role in the formation of purine nucleotides via the purine salvage pathway by catalyzing the conversion of hypoxanthine. This gene was also selected for the ER Map^®^/ER Grade^®^ panel as being involved in biological processes occurring in the preimplantation endometrium [11]. The purine hypoxanthine plays important roles in the regulation of oocyte maturation and early embryonic development. HPRT deficiency in rats disrupts early embryonic development and causes infertility in females [40]. This finding suggests the presence of metabolic disturbances in RIF associated with a thin endometrium. However, HPRT deficiency is a rare disorder; therefore, the decrease in this enzyme’s activity in receptivity disorders needs to be studied.

This study showed that patients with RIF and PNL had significant (*p* < 0.05) inverse moderate correlations. In patients with RIF, significant inverse moderate correlations were observed between HAND2 and TSH, HAND2 and fibrinogen, VEGFB and prolactin, and VEGFB and TSH. In patients with PNL, significant inverse moderate correlations were found between CXCL1 and the prothrombin time and between MMP10 and the activated partial thromboplastin time, and a direct moderate correlation was found between CXCL1 and the activated partial thromboplastin time. Thus, decreases in the levels of the above genes were associated with increases in the levels of pituitary hormones and the activation of the hemostasis system.

This study confirmed that the RIF and RPL populations are extremely heterogeneous. Despite the similarities of the risk factors, the gene expressions differ. However, the prognostic potentials of the *IL-15* and *INFG* genes and their combinations for developing therapeutic approaches in women with RIF and thin endometria were shown. We assessed the ability to predict IVF failure by monitoring the transcript levels of the three genes. The transcripts alone showed modest results in the area of the threshold between “fair” and “poor” (with an AUC of approximately 0.7); however, the combination of IL15 + HPRT1 reached the threshold of a “good” classifier (AUC = 0.8) according to Muller et al.’s grading system (2005) [24].

This study’s main limitation was the small sample size, which should be considered in the future. Furthermore, stratification by clinical forms is recommended. Further studies are needed to confirm the observed changes in gene expressions at the proteome and metabolome levels.

## 5. Conclusions

In summary, the pathophysiology of non-receptive endometria in women with RIF and RPL involves a complex interplay of cytokine imbalances, defective decidualization, impaired angiogenesis, and immune system dysfunction. Understanding these mechanisms is critical for developing targeted therapeutic strategies to improve reproductive outcomes. This study revealed decreases in the expressions of the *IL-15*, *INFG*, and *HPRT1* genes in the thin endometria of RIF patients compared with women with RPL. These data contribute to understanding the processes in a thin endometrium, leading to implantation failures during IVF, and are of practical significance for clinicians for the differentiated prescription of immunomodulatory therapy in patients undergoing ART programs.

## Figures and Tables

**Table 1:** Demographic and clinical characteristics of the study groups.

Characteristic	RIF Patients (*n* = 20)	RPL Patients (*n* = 19)
Age (years): Mean ± SD	34.65 ± 5.29	33.37 ± 5.22
BMI (kg/m^2^): Mean ± SD	23.36 ± 4.24	25.69 ± 4.79
Endometrium thickness (mm): Mean ± SD	6.51 ± 1.27	6.13 ± 1.86
Fibrinogen, g/L: Mean ± SD	3.05 ± 0.51	3.10 ± 0.23
Prothrombin index, %: Mean ± SD	99.16 ± 8.22	97.57 ± 6.49
Prothrombin time, sec: Mean ± SD	13.04 ± 2.35 *	14.69 ± 1.78
Activated partial thromboplastin time, sec: Mean ± SD	34.04 ± 3.61	32.10 ± 2.20
International normalized ratio: Mean ± SD	1.04 ± 0.12	1.21 ± 0.14
Anti-Mullerian hormone, ng/mL: Mean ± SD	2.60 ± 1.03	2.64 ± 0.32
Luteinizing hormone, mIU/mL: Mean ± SD	8.34 ± 2.13	11.24 ± 3.26
Follicle-stimulating hormone, mIU/mL: Mean ± SD	6.19 ± 1.43	6.00 ± 1.07
Prolactin, mIU/L: Mean ± SD	325.0 ± 152.4	333.6 ± 61.6
Thyroid-stimulating hormone, mIU/mL: Mean ± SD	2.84 ± 0.74	nd
Chronic endometritis, yes/no	20/0	16/3
Chronic Salpingo-oophoritis, yes/no	10/10	10/9
Pelvic organ surgeries, yes/no	10/10	6/13
Endometriosis, yes/no	5/15	4/15
Uterine fibroids, yes/no	1/19	3/16
Polyps, yes/no	1/19	4/15

* *p* < 0.05; nd—no data.

**Table 2:** Comparative gene expression statistics between RIF and RPL in women.

Gene	Ct (Mean ± SD)		ΔCt (Mean ± SE)		ΔΔCt (95% CI) Log2 Fold Change	*p* Value
	RIF	RLP	RIF	RLP		
*C4BPA*	30.51 ± 3.75	29.69 ± 3.29	−8.20 ± 0.61	−9.65 ± 0.38	0.99 (−0.06; 2.13)	0.059
*CXCL1*	28.19 ± 4.85	27.84 ± 4.27	−5.53 ± 0.65	−6.82 ± 0.29	0.83 (−0.33; 1.97)	0.175
*HAND2*	24.36 ± 4.29	22.90 ± 3.52	−1.69 ± 0.44	−1.88 ± 0.17	0.35 (−0.24; 1.00)	0.235
*HPRT1*	25.59 ± 3.81	24.77 ± 4.23	−2.92 ± 0.29	−3.75 ± 0.13	**0.57 (0.01; 1.54)**	**0.041**
*IFNG*	33.68 ± 2.49	33.58 ± 2.85	−12.27 ± 0.48	−13.52 ± 0.36	**1.24 (0.01; 2.43)**	**0.046**
*IL15*	26.54 ± 3.63	25.89 ± 3.89	−3.88 ± 0.28	−4.87 ± 0.22	**0.92 (0.10; 1.65)**	**0.023**
*IL8*	28.93 ± 4.19	28.16 ± 4.00	−6.26 ± 0.67	−7.55 ± 0.54	1.09 (−0.04; 2.55)	0.070
*MMP10*	32.07 ± 3.89	29.76 ± 5.55	−9.41 ± 0.86	−8.74 ± 0.66	−0.04 (−2.70; 1.81)	0.945
*TNC*	27.24 ± 4.15	25.07 ± 4.49	−4.57 ± 0.45	−4.04 ± 0.40	−0.58 (−1.45; 0.78)	0.396
*VEGFB*	25.64 ± 3.46	24.20 ± 3.41	−2.97 ± 0.19	−3.18 ± 0.21	0.34 (−0.23; 0.77)	0.214
*GAPDH*	22.90 ± 4.09	21.24 ± 4.45	-	-	-	-
*YWHAZ*	22.43 ± 3.47	20.81 ± 3.89	-	-	-	-

Note: significant differences (*p* < 0.05) are in bold.

**Table 3:** Detected correlations between gene expression levels and clinical and laboratory parameters.

Group	Parameter 1	Parameter 2	Spearman’s Rho Value	*p* Value
RPL group	*CXCL1*	Prothrombin time	−0.456	0.04959
	*CXCL1*	Activated partial thromboplastin time	0.587	0.0083
	*MMP10*	Activated partial thromboplastin time	−0.494	0.0317
RIF group	*VEGFB*	Prolactin	−0.446	0.049
	*VEGFB*	Thyroid-stimulating hormone	−0.526	0.017
	*HAND2*	Thyroid-stimulating hormone	−0.671	0.001
	*HAND2*	Fibrinogen	−0.465	0.038

**Table 4:** ROC analysis results.

Potential Markers and Combinations	AUC (95% CI)	Optimal Cut-Off Point	Sensitivity (95% CI)	Specificity (95% CI)
*IL15*	0.713 (0.550–0.876)	−4.091	0.800 (0.563–0.943)	0.579 (0.335–0.797)
*IFNG*	0.701 (0.516–0.886)	−13.238	0.667 (0.410–0.867)	0.750 (0.476–0.927)
*HPRT1*	0.692 (0.505–0.879)	−3.568	0.900 (0.683–0.988)	0.632 (0.384–0.837)
*IL15 + IFNG*	0.722	-	0.667	0.688
*IL15 + HPRT1*	0.800	-	0.850	0.684
*IFNG + HPRT1*	0.753	-	0.778	0.750
*IL15 + IFNG + HPRT1*	0.778	-	0.889	0.688

## Data Availability

Experimental and clinical–pathological data are available.
